# Avoiding Stigma in Dementia Prevention Discourse: Understanding Barriers and Harnessing Strengths.

**DOI:** 10.1093/geroni/igaf122.296

**Published:** 2025-12-31

**Authors:** Nikki-Anne Wilson

**Affiliations:** University of New South Wales Sydney/Neuroscience Research Australia, Kensington, New South Wales, Australia

## Abstract

Recent decades have seen exponential growth in research on modifiable risk factors for dementia across the lifespan. We now have an unprecedented understanding of ways to support brain health, particularly the need for healthy eating and physical activity which are commonly referred to as “lifestyle choices”. Individualistic lifestyle modifications remain the leading dementia prevention focus and their importance should not be understated. We caution against, however, a potential short-sightedness regarding the bounds of individual responsibility and the need for a broader understanding of individual and socio-economic barriers to instigating positive health behaviors. People living with dementia already experience stigma and we know that negative health outcomes do occur for people with other non-communicable “lifestyle diseases” e.g., diabetes. Moving forward, we call for balance in dementia prevention discourse capturing both the complexities of etiology and a more inclusive approach to risk-reduction which addresses health inequities. This requires both population-level interventions and multidisciplinary individual support. Further, given our broadening understanding of psychosocial factors such as depression and social isolation as contributing to dementia risk, we suggest that a greater focus on strengths-based approaches to building social and psychological wellbeing may be beneficial. Our aim is not to detract from work which has come before nor undermine the continued importance of empowering individuals to make positive health changes. Rather, we put forward the need for a more considered and inclusive approach to the investigation and dissemination of dementia risk-reduction which avoids inadvertently increasing stigma through overly simplistic representations of a complex clinical syndrome.

